# *Capicua* (*CIC*) mutations in gliomas in association with MAPK activation for exposing a potential therapeutic target

**DOI:** 10.1007/s12032-023-02071-0

**Published:** 2023-06-08

**Authors:** Sourat Darabi, Joanne Xiu, Timothy Samec, Santosh Kesari, Jose Carrillo, Sonikpreet Aulakh, Kyle M. Walsh, Soma Sengupta, Ashley Sumrall, David Spetzler, Michael Glantz, Michael J. Demeure

**Affiliations:** 1 Hoag Family Cancer Institute, Newport Beach, CA USA; 2 Caris Life Sciences, Phoenix, AZ USA; 3 Pacific Neuroscience Institute, Providence Saint John’s Health Center, Santa Monica, CA USA; 4 West Virginia University, Morgantown, WV USA; 5 Duke University School of Medicine, Durham, NC USA; 6 University of Cincinnati College of Medicine, Cincinnati, OH USA; 7 Levine Cancer Institute, Charlotte, NC USA; 8 Penn State Hershey Medical Center, Hershey, PA USA; 9 Translational Genomics Research Institute, Phoenix, AZ USA

**Keywords:** *Capicua*, MAPK, Glioma, Targeted medicine, Molecular oncology, Clinical oncology

## Abstract

Gliomas are the most prevalent neurological cancer in the USA and care modalities are not able to effectively combat these aggressive malignancies. Identifying new, more effective treatments require a deep understanding of the complex genetic variations and relevant pathway associations behind these cancers. Drawing connections between gene mutations with a responsive genetic target can help drive therapy selections to enhance patient survival. We have performed extensive molecular profiling of the Capicua gene (*CIC)*, a tumor and transcriptional suppressor gene, and its mutation prevalence in reference to MAPK activation within clinical glioma tissue. *CIC* mutations occur far more frequently in oligodendroglioma (52.1%) than in low-grade astrocytoma or glioblastoma. *CIC*-associated mutations were observed across all glioma subtypes, and MAPK-associated mutations were most prevalent in *CIC* wild-type tissue regardless of the glioma subtype. MAPK activation, however, was enhanced in *CIC*-mutated oligodendroglioma. The totality of our observations reported supports the use of *CIC* as a relevant genetic marker for MAPK activation. Identification of *CIC* mutations, or lack thereof, can assist in selecting, implementing, and developing MEK/MAPK-inhibitory trials to improve patient outcomes potentially.

**Supplementary Information:**

The online version contains supplementary material available at 10.1007/s12032-023-02071-0.

## Background

In 2022, neurological cancers were estimated to exceed 25,000 cases and result in over 18,000 deaths in the USA [1]. Around 80% of these deaths were due to malignant gliomas, the most common primary brain cancer in adults [2]. Diffuse adult gliomas are primarily categorized as either astrocytomas or oligodendrogliomas. Lower-grade (WHO 2–3) astrocytomas encompass nearly 10% of gliomas, while Grade 4 astrocytomas, also known as glioblastoma multiforme (GBM), account for 55% of all gliomas. These highly aggressive tumors exhibit one of the lowest survival rates of all cancers, with a 10-year survival of just 2.6% [3, 4]. Five-year survival rates for grades 2 and 3 astrocytomas range between 30 and 50%, respectively [5]. Oligodendrogliomas, defined by the presence of IDH1/2 mutation and co-occurring loss of chromosomes 1p and 19q, make up < 7% of adult diffuse glioma diagnoses. They are relatively slow-growing tumors, with a much better prognosis if discovered at an early age [5–7].

The *Capicua* (*CIC*) gene is a negative regulator of the mitogen-activated protein kinase (MAPK) signaling pathway [8]. Originally studied in Drosophila*, CIC* has been recognized as a prospective tumor suppressor gene that mediates cell proliferation and mobility [9]. Located on chromosome 19q, *CIC* is mutated in nearly 70% of oligodendrogliomas, contributing to poor patient prognosis [10, 11]. *CIC* knockout, deletion, and mutations have been shown to influence the formation of other cancers, including T-cell lymphoblastic lymphoma, Ewing sarcoma, and metastasis of epidermal growth factor receptor (*EGFR*) inhibitor-resistant lung adenocarcinoma [8, 12–14]. With respect to glioma, studies have shown frequent mutation of *CIC* in oligodendroglioma but very low mutation frequency in GBM and lower-grade astrocytoma [9]. However, studies performed in *Drosophila* suggest a relationship between *CIC* mutations and enhanced MAPK pathway signaling [15]. Nearly 88% of gliomas exhibit MAPK pathway alterations, affecting gene-regulated cell processes, including invasion and proliferation, and playing a role in growth and invasion [16]. Relationships between *CIC* and its expression levels, MAPK activation, and mutations within both genes can modulate apoptotic activity in malignant cells and enhance survival under hypoxic conditions [17]. Our work seeks to establish a relationship between *CIC* and MAPK in glioma, as well as generate an understanding of the implications of *CIC* and MAPK gene expression and mutation levels as they pertain to patient survival and response to current standards of care, possibly introducing *CIC* as a novel gene target for MAPK-directed therapies.

## Methods

### Next-generation sequencing (NGS)

NGS was performed on genomic DNA isolated from formalin-fixed paraffin-embedded (FFPE) tumor samples by a commercial CAP/CLIA lab using the NextSeq or NovaSeq 6000 platforms (Illumina, Inc., San Diego, CA). For NextSeq-sequenced tumors, a custom-designed SureSelect XT assay was used to enrich 592 whole-gene targets (Agilent Technologies, Santa Clara, CA). For NovaSeq-sequenced tumors, a panel of more than 700 clinically relevant genes was sequenced at high coverage and high read-depth, along with another panel designed to enrich for additional > 20,000 genes at a lower depth. All variants were detected with > 99% confidence based on allele frequency and amplicon coverage, with an average sequencing depth of coverage of > 500 × and an analytic sensitivity of 5%. Variant testing included single-nucleotide variants (SNV) and insertions and deletions (INDEL) on a panel of 720 genes. Copy number alteration (CNA) analysis was performed on over 400 genes and a 592-gene panel was used for all mutations. Prior to molecular testing, tumor enrichment was achieved by harvesting targeted tissue using manual microdissection techniques. For DNA sequencing, 20% of minimum tumor content was required. Genetic variants identified were interpreted by board-certified molecular geneticists and categorized as ‘pathogenic,’ ‘likely pathogenic,’ ‘variant of unknown significance,’ ‘likely benign,’ or ‘benign,’ according to the American College of Medical Genetics and Genomics (ACMG) standards. When assessing mutation frequencies of individual genes, ‘pathogenic’ and ‘likely pathogenic’ were counted as mutations [18]. The copy number alteration (CNA) of each exon is determined by calculating the average depth of the sample along with the sequencing depth of each exon and comparing this calculated result to a pre-calibrated value.

TMB was measured by counting all non-synonymous missense, nonsense, in-frame insertion/deletion, and frameshift mutations found per tumor that had not been previously described as germline alterations in dbSNP151, Genome Aggregation Database (gnomAD) databases or benign variants identified by Caris geneticists. A cutoff point of ≥ 10 mutations per MB was used based on the KEYNOTE-158 pembrolizumab trial which showed that patients with a TMB of ≥ 10 mt/MB across several tumor types had higher response rates than patients with a TMB of < 10 mt/MB [19].

### Whole-transcriptome sequencing

Gene fusion detection was performed on mRNA isolated from a formalin-fixed paraffin-embedded tumor sample using the Illumina NovaSeq platform (Illumina, Inc., San Diego, CA) and Agilent SureSelect Human All Exon V7 bait panel (Agilent Technologies, Santa Clara, CA). FFPE specimens underwent pathology review to diagnose percent tumor content and tumor size; a minimum of 10% of tumor content in the area for microdissection was required to enable enrichment and extraction of tumor-specific RNA. A Qiagen RNA FFPE tissue extraction kit, was used for extraction, and the RNA quality and quantity were determined using the Agilent TapeStation. Biotinylated RNA baits were hybridized to the synthesized and purified cDNA targets and the bait-target complexes were amplified in a post-capture PCR. The resultant libraries were quantified and normalized, and the pooled libraries are denatured, diluted, and sequenced; the reference genome used was GRCh37/hg19 and analytical validation of this test demonstrated ≥ 97% Positive Percent Agreement (PPA), ≥ 99% Negative Percent Agreement (NPA) and ≥ 99% Overall Percent Agreement (OPA) with a validated comparator method. For gene expression, the whole transcriptome from patients was sequenced to an average of 60-M reads. Raw data were demultiplexed by Illumina Dragen BioIT accelerator, trimmed, counted, PCR duplicates removed, and aligned to human reference genome hg19 by STAR aligner. For transcript counting, transcripts per million numbers was generated using the Salmon expression pipeline [20]. Immune cell fraction was calculated by quanTIseq [21].

### PyroSeq

MGMT promoter methylation was evaluated by pyrosequencing. DNA extraction from paraffin-embedded tumor samples was performed for subsequent pyrosequencer-based analysis of 5 CpG sites (CpGs 74–78). All DNA samples underwent a bisulfite treatment and were PCR amplified with primers specific for exon 1 of MGMT (GRCh37/hgl9—chr10: 131,265,448- 131,265,560). The methylation status of PCR-amplified products is determined using the PyroMark system (Qiagen, Germantown, MD). Samples with ≥ 7% and < 9% methylation are considered to be equivocal or gray zone results.

### Immunohistochemistry

Immunohistochemistry (IHC) of MLH1, M1 antibody; MSH2, G2191129 antibody; MSH6, 44 antibody; and PMS2, EPR3947 antibody were performed on FFPE sections of glass slides. Slides were stained using automated staining techniques, per the manufacturer’s instructions (Ventana Medical Systems, Inc. Tucson, AZ) and were optimized and validated per CLIA/CAP and ISO requirements. Staining was scored for intensity (0 = no staining; 1 + = weak staining; 2 + = moderate staining; 3 + = strong staining) and staining percentage (0–100%). The complete absence of protein expression of any of the 4 proteins tested (0 + in 100% of cells) was considered deficient MMR. A board-certified pathologist evaluated all IHC results independently.

### MSI/MMR status

A combination of multiple test platforms was used to determine the MSI or MMR status of the tumors profiled, including fragment analysis (FA, Promega, Madison, WI), IHC (see IHC method), and NGS (7000 target microsatellite loci were examined and compared to the reference genome hg19 from the University of California). The three platforms generated highly concordant results, as previously reported. In the rare cases of discordant results, the MSI or MMR status of the tumor was determined in the order of IHC, FA, and NGS [22].

### MPAS

MAPK activation score (MPAS) score was calculated based on the TPM values of RNA expression of 10 genes (*SPRY2*, *SPRY4*, *ETV4*, *ETV5*, *DUSP4*, *DUSP6*, *CCND1*, *PHLDA1*, *EPHA2*, and *EPHA4*) using a previously reported algorithm as a transcriptomic indicator of MAPK pathway activation [23].

### CODEai

Real-world overall survival (rwOS) information was obtained from insurance claims data and calculated from first of treatment time to last of treatment time (TOT). Kaplan–Meier estimates were calculated for molecularly defined patient cohorts. Significance was determined as *p* values of < 0.05.

## Results

### CIC demographic and mutation rates in glioma subtypes

A total of 7341 glioma tumor samples were classified based on histology subtypes and characterized for *CIC* mutation status (Table 1). In total, 296 (4.0%) of gliomas were *CIC*- mutated, including glioblastoma (0.6%), astrocytoma (1.7%), oligodendroglioma (52.1%), and mixed/unclear typing (10.3%). Of the 19 *CIC*-mutated astrocytoma samples, 10 were grade 3 and 9 were grade 2. None of the pilocytic/grade 1 astrocytomas were *CIC* mutated. Of the 202 *CIC*-mutated oligodendroglioma samples, 93 were grade 3 and 109 were grade 2 (Table 2). In all gliomas analyzed together, *CIC* mutations were associated with younger age (46 yrs. vs. 58 yrs.) and female gender (50% vs. 41%). These differences were significant, however, only in GBM tumors (median age 49 yrs. vs. 60 yrs., *p* ≤ 0.00019; female prevalence 58% vs. 40%, *p* ≤ 0.037; Table 1). Additionally, the association with age was reversed in the oligodendroglioma subset, with *CIC*-mutated tumors being found in significantly older patients (median age 45.5 yrs. vs. 42 yrs., *p* ≤ 0.014).

**Table 1:** Demographic patient data by *Capicua* (*CIC*) mutation status and glioma subtype

	CIC Mut	CIC WT	*p*-value
**All Gliomas**			
Count (N)	296	7169	
Median age [range] (N)	46 [20—78] (296)	58 [0—89] (7169)	5.84E-22
Male	49.7% (147/296)	59.4% (4257/7169)	0.000863927
Female	50.3% (149/296)	40.6% (2912/7169)	
**Oligodendroglioma**			
Count (N)	202	186	
Median age [range] (N)	45.5 [21—77] (202)	42 [5—78] (186)	0.014423866
Median TMB [range] (N)	4.0 [1.0—272.0] (197)	3.0 [0.0—95.0] (181)	0.000753571
Male	50.0% (101/202)	58.1% (108/186)	0.111398203
Female	50.0% (101/202)	41.9% (78/186)	
**Astrocytoma**			
Count (N)	19	1094	
Median age [range] (N)	41 [24—75] (19)	41 [0—89] (1094)	0.516739491
Female	42.1% (8/19)	41.7% (456/1094)	0.970399172
Male	57.9% (11/19)	58.3% (638/1094)	
**GBM**			
Count (N)	33	5310	
Median age [range] (N)	49 [20—78] (33)	60 [2— 89] (5310)	0.000197057
Male	42.4% (14/33)	60.2% (3197/5310)	0.03755952
Female	57.6% (19/33)	39.8% (2113/5310)

**Table 2:** *CIC* mutation rates within each glioma subtype

Tumor type	Mut	WT	Total	Mut %
Astrocytoma	19	1094	1113	1.7
Anaplastic/grade 3/high grade	10	551	561	1.8
Diffuse/grade2/low grade	9	447	456	2.0
Pilocytic/grade 1	0	96	96	0.0
Ependymoma	0	13	13	0.0
Ganglioglioma	0	37	37	0.0
GBM	33	5310	5343	0.6
Gliosarcoma	0	124	124	0.0
Glioneuronal	0	11	11	0.0
Oligodendroglioma	202	186	388	52.1
Anaplastic/grade 3/high grade	93	93	186	50.0
Diffuse/grade2/low grade	109	93	202	54.0
Pleomorphic xanthoastrocytoma	0	29	29	0.0
Unclear	42	365	407	10.3
Total	296	7169	7341	4.0

### CIC mutations are associated with increased alteration rates of glioma-relevant genes but Not MAPK-associated genes

**Fig. 1:**
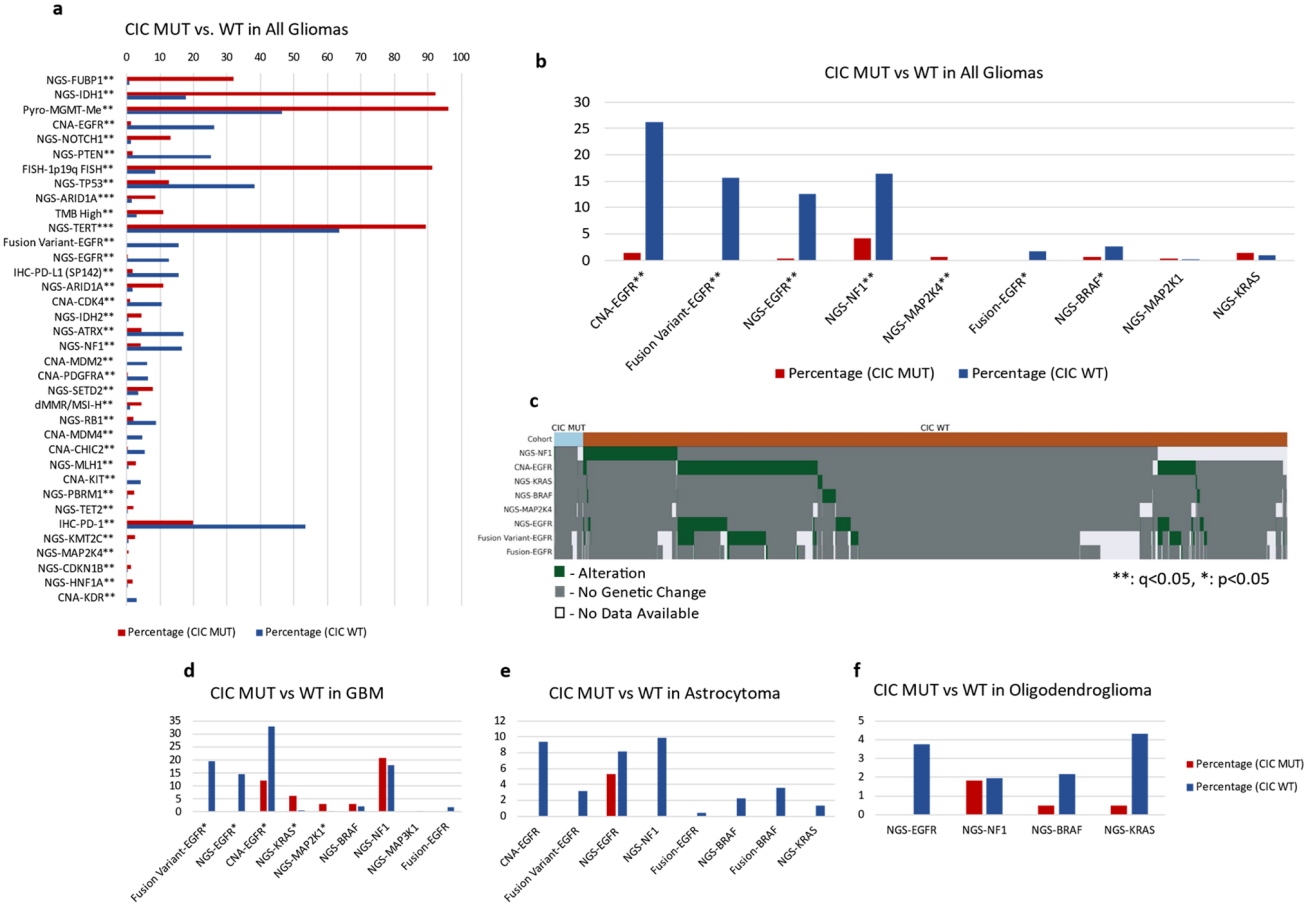
Gene mutation rates are higher in *CIC*-mutant tissue except for MAPK-related gene in all glioma and all glioma subtypes. A selection of genes and signatures from the array of analysis techniques relevant to tumorigenesis exhibiting mutation relative to *CIC* mutation or wild-type status (**a**). Similarly, MAPK-relevant gene mutation status was also characterized (**b**) and quantified by *CIC* mutation status; Red = *CIC* MUT, Blue = *CIC* WT. MAPK-relevant genes shown via oncoprint by *CIC* mutation status as *CIC* mutant (light blue), *CIC* wild-type (orange), genetic alteration (green), no genetic change (gray), or no data available (white) (**c**). Mutation rates of MAPK-associated genes in GBM (**d**), astrocytoma (**e**), and oligodendroglioma (**f**) by *CIC* mutation status; Red = *CIC* MUT, Blue = *CIC* WT. *q < 0.05, **q < 0.01, and ***q < 0.001

Whole-Transcriptome Sequencing (WTS), Next-Generation Sequencing (NGS), and immunohistochemistry (IHC) were performed on FFPE tissue samples to identify any molecular alterations associated with CIC mutation status. As shown in Fig. 1A, CIC-mutated tumor samples in all gliomas exhibited significant increases in mutation rates of oncogenic drivers, including *FUBP1*, *NOTCH1*, *ARID1A*, *IDH2*, *MLH1*, *TET2*, *KMT2C*, and *CDKN1B*. In addition, increased rates of 1p19q codeletion, *TERT* promoter mutations, *MGMT* promoter methylation, and dMMR/MSI were observed in *CIC*-mutated samples.

Conversely, only MAPK pathway-activating alterations were increased in wild-type *CIC* tumors compared to *CIC*-mutated tumors. Significant increases of *EGFR* alterations (including activating mutations, fusions, and the *EGFRvIII* variant) and *NF1* and *BRAF* mutations were observed in *CIC* wild-type samples (Fig. 1B). The only significant increase in gene mutation associated with *CIC* mutation compared to *CIC* wild-type was *MAP2K4* (Fig. 1B). Further genetic alterations associated with CIC mutation status is shown in the OncoPrint in Fig. 1C. Significant genetic alterations, shown in green, were observed in *CIC* wild-type samples, while few alterations were observed in *CIC*-mutant samples, confirming the largely mutually exclusive pattern of *CIC* mutation with other MAPK pathway changes.

**Fig. 2:**
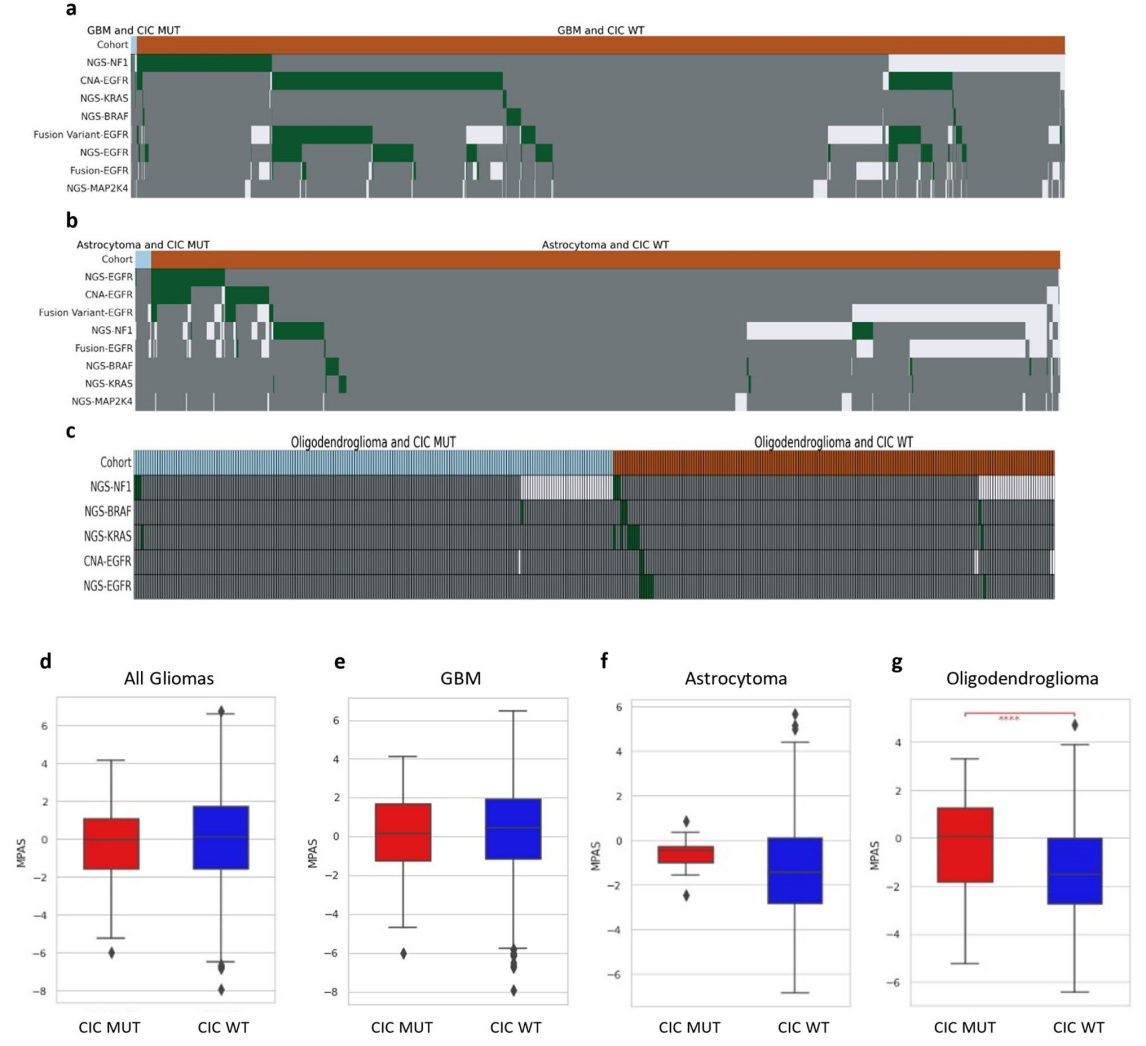
MAPK-relevant genetic alterations are more frequently observed in *CIC*-mutated GBM and astrocytoma with MAPK Activation Score (MPAS) significantly lower for *CIC* wild-type oligodendroglioma. Oncoplot projections depict MAPK-relevant gene alterations (green), no gene alterations (gray), or no data (white) for GBM (**a**), astrocytoma (**b**), and oligodendroglioma (**c**) by *CIC* mutation status. Quantification of MAPK activation was calculated via expression profiles of 10 MAPK-associated genes, yielding the MPAS. No significant activation differences were observed between *CIC*-mutant (red) and *CIC* wild-type (blue) tissues in all glioma (**d**), GBM (**e**), or astrocytoma (**f**). Oligodendroglioma (**g**) exhibited lower MPAS in *CIC* wild-type tissue. ****q < 0.0001

Our data across GBM (Fig. 1D), astrocytoma (Fig. 1E), and oligodendroglioma (Fig. 1F) show MAPK-associated gene alterations between *CIC*-mutated glioma and *CIC* wild-type tumors. Specifically, *CIC* wild-type tissue exhibits higher mutation rates of MAPK-associated genes across all glioma subtypes, including more valent *EGFR* mutation, amplification, *EGFRvIII* variant, and *EGFR* fusion. When the relative relationship of MAPK-associated alterations and *CIC* mutations are examined on OncoPrints (Fig. 2A–C), GBM MAPK-associated gene alterations (Fig. 2A) were shown to be quite prominent in *CIC* wild-type tumor samples, while *CIC*-mutated tumors had few alterations in *NF1*, *KRAS*, and *EGFR*. Similarly, astrocytoma (Fig. 2B) and oligodendroglioma (Fig. 2C) exhibited a much higher rate of MAPK-relevant genetic alterations in *CIC* wild-type tumors where *CIC*-mutant tumors only displayed few alterations in *EGFR*, *NF1*, and *KRAS*.

Quantitatively capturing the MAPK pathway association with *CIC* mutation status via the MAPK Pathway Activity Score (MPAS) scoring method may assist in prognostic evaluations and biomarker associations in various cancer types [23]. Current literature has not yet described MPAS scoring in glioma in association with *CIC* mutation status. As shown in Fig. 2D–G, significant differences in MAPK activation in *CIC*-mutant tumors as compared to wild-type only appear in oligodendroglioma, but is not seen in GBM, astrocytoma, or when all three histological subtypes are combined.

### GBM, astrocytoma, and oligodendroglioma oncogenic profiles are enhanced in CIC-mutated samples versus CIC wild-type samples

**Fig. 3:**
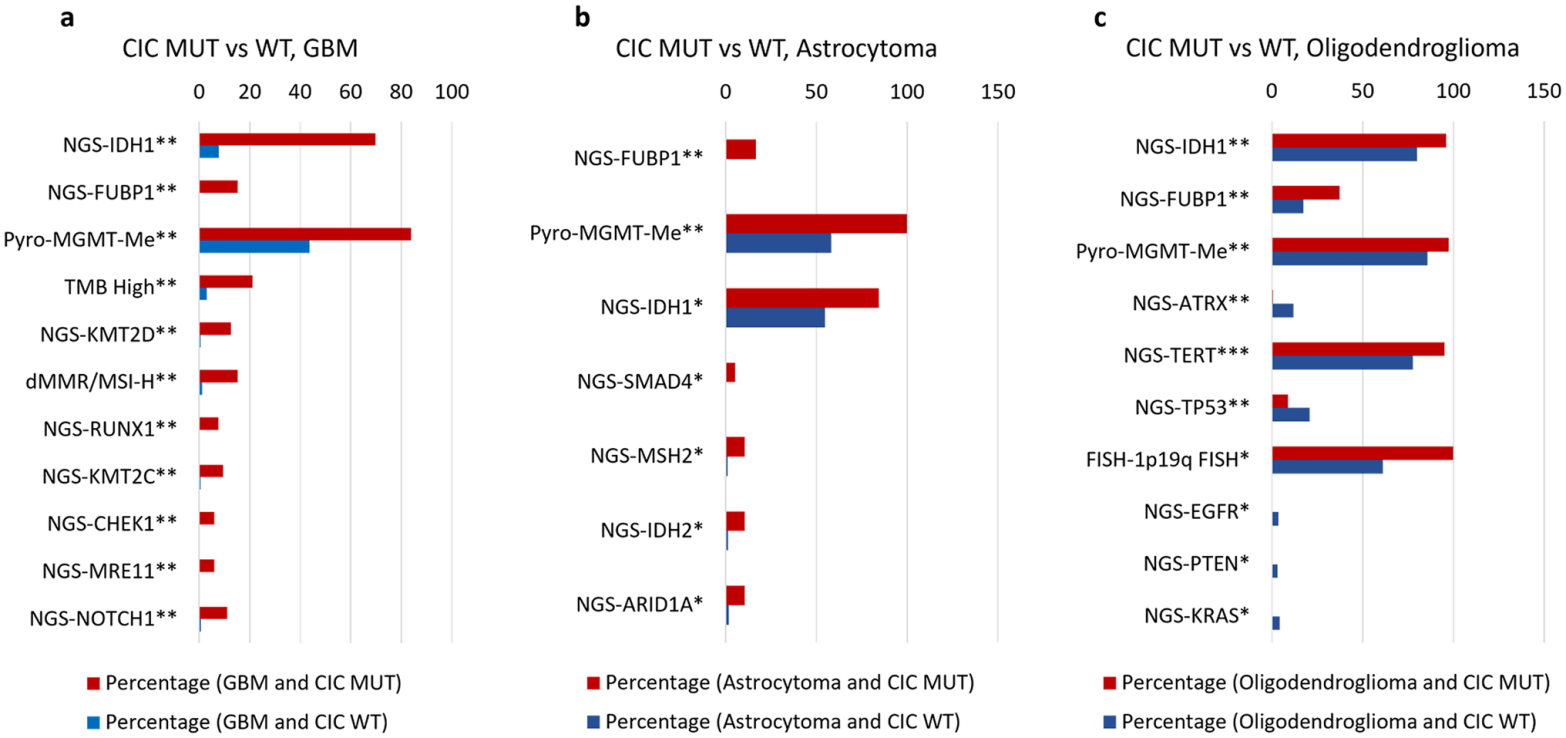
Genetic alterations in glioma subtypes are more prevalent in *CIC*-mutant tissue. Mutation rates of oncologic-related genes and signatures quantified by NGS, Whole-Exome Sequencing (WES) and WTS for GBM (**a**), astrocytoma (**b**), and oligodendroglioma (**c**) by *CIC* mutation status. *q < 0.05, **q < 0.01, and ***q < 0.001

The most prominent glioma subtypes, GBM, astrocytoma, and oligodendroglioma, were individually analyzed to characterize molecular differences between *CIC* mutants and their *CIC* wild-type counter parts. As shown in Fig. 3, a majority of oncogenic driver-related genetic alterations were observed in *CIC*-mutant tumors in GBM (Fig. 3A), astrocytoma (Fig. 3B), and oligodendroglioma (Fig. 3C). Two similarities among the three subtypes were characterized: high prevalence of *MGMT* promoter methylation, *FUBP1* mutation, and *IDH1* mutation associated with *CIC* mutants. Numerous differences, however, were apparent, including increased TMB-high and dMMR/MSI-H prevalence in GBM, and a high rate of *TERT* promoter mutation and 1p19q codeletion in oligodendroglioma. An increased *TP53* mutation rate was observed in *CIC* wild-type oligodendrogliomas. These results confirm previous reports of significant genetic differences between glioma subtypes and reveal tumor type-specific molecular associations with *CIC* [24].

### CIC mutation is positively correlated with patient survival in oligodendroglioma and temozolomide (TMZ)-treated glioma

**Fig. 4:**
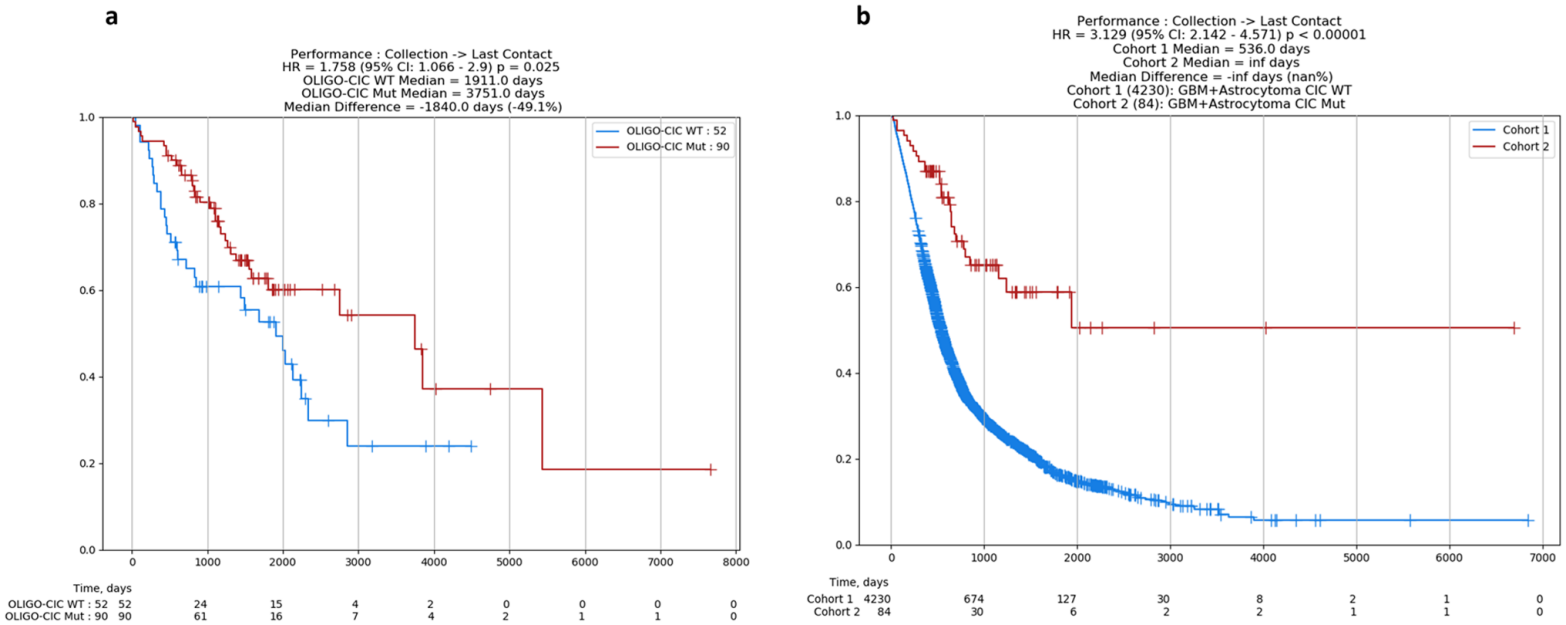
Glioma patient survival is enhanced with the presence of *CIC* mutation in comparison to *CIC* wild-type. CODEai survival analysis on oligodendroglioma (**a**) and astrocytoma and GBM patients (grouped) (**b**) by mutated *CIC* (red) status and wild-type *CIC* (blue) status

Patients harboring both *CIC* mutation and *CIC* wild-type oligodendroglioma, astrocytoma, and GBM were monitored for over one year following diagnosis to last contact. In oligodendroglioma (Fig. 4A), patients with *CIC* mutations (90) survived a median 3751 days, while *CIC* wild-type patients had a median survival of 1911 days. The hazard ratio (HR) between the two groups was 1.758 and a statistically significant difference of median survival of 1840 days yielding *p* ≤ 0.025. Similarly, as shown in Fig. 4B, patients with either astrocytoma or GBM and *CIC* wild-type status were grouped into cohort 1 and patients with either astrocytoma or GBM and *CIC* mutations were grouped into cohort 2. Cohort 1 was followed to a median 536-day survival and cohort 2 did not show a quantifiable end survival date. Because of this, the median difference between the two cohorts was characterized as ‘infinite’ and a *p* ≤ 0.0001. Additionally, patients who were treated with a TMZ regimen were monitored from first dose of TMZ until last contact. Similar to patients who were not undergoing TMZ treatment, both the oligodendroglioma and combined GBM + astrocytoma cohorts with mutated *CIC* exhibited longer survival periods (Supp. Figure 1). Specifically, oligodendroglioma patients currently on TMZ therapy with *CIC* mutation survived a median of 2296 days, while patients with wild-type *CIC* treated with TMZ survived a median of 907 days (*p* = 0.002, HR = 2.865) (Supp. Figure 1). In GBM + astrocytoma patients, those with mutated *CIC* currently on TMZ therapy survived for a median of 1874 days and those patients without *CIC* mutation survived a median of 602 days (*p* ≤ 0.001, HR = 2.688) (Supp. Figure 1).

## Discussion

Gliomas are difficult to treat and usually result in patient demise within five years of diagnosis [1]. Identification of novel biomarkers to provide clarity on possible patient prognosis and therapeutic response can help create a set of reliable indicators for selecting appropriate therapies [25]. Our study analyzed the prospect of using the *CIC* gene as an indication of MAPK upregulation within glioma subtypes as well as exploring the underlying genetic landscape that coincides with *CIC* mutations. This work has shown that the mutation of *CIC* in glioma was mutually exclusive to MAPK genomic driver alterations, specifically *NF1*, *BRAF*, and *KRAS*. Further, characterization of MAPK activation via MPAS revealed higher activation for mutated *CIC* in comparison to wild-type *CIC*. This observation is in line with other studies that discuss the negative correlation between unaltered *CIC*, MAPK activation, and MAPK driver expression [8, 13, 14, 26].

The current standard of care for all glioma subtypes is tumor resection, if possible [27]. Further decisions on therapeutic modalities are then based on the biomarkers of the tumor and, according to the 5th Edition of the World Health Organization Classification of Tumors of the Central Nervous System (WHO CNS5), particularly the mutational status of isocitrate dehydrogenase (*IDH*) and the methylation status of methylguanine methyltransferase (*MGMT*) gene [28]. The National Comprehensive Cancer Network (NCCN) utilizes the status of *IDH1* and *IDH2*, *MGMT* methylation, codeletion of 1p/19q, and *ATRX* mutation to drive therapeutic decisions [29]. Low-grade gliomas are commonly treated with radiotherapy and a combination of concurrent or adjuvant TMZ chemotherapy and/or procarbazine and vincristine dependent on the patient-specific molecular markers [28–30]. Patients with high-grade glioma, specifically GBM, are usually treated with local radiotherapy and combinatorial TMZ chemotherapy [28, 31]. Unfortunately, most patients receiving these therapies either do not respond or experience recurrence, commonly resulting in death. Numerous clinical trials have been introduced attempting to improve the treatment of glioma, including growth factor receptor inhibition, both *EGFR* and fibroblast (*FGFR*); apoptotic pathway regulation; angiogenic targets; and several immunotherapeutic approaches [32–39]. Though some trials have had success in improving overall survival [32–35, 39], limitations arise in matching targeted therapies to the molecular profile of glioma subtypes.

Here, we describe the prospect of the *CIC* gene as a target to evaluating tumorigenesis and predicting MAPK-associated therapeutic selections. Previous literature has reported *CIC* expression and mutation patterns in gliomas with nearly 40% of oligodendroglioma tumors harboring *CIC* alterations [10, 40]. Further, these alterations in oligodendroglioma along with loss of *FUBP1* expression have been shown to be potential markers of rapid recurrence [10]. We have confirmed these observations by showing 50.2% mutation rate in oligodendroglioma, 51.4% mutation rate in anaplastic, high-grade oligodendroglioma, and 49.0% mutation rate in diffuse, low-grade oligodendroglioma. Glioblastoma and astrocytoma both exhibited very low mutation rates at 0.6% and 1.9%, respectively. Though mutation rates vary significantly across histological subtypes, each subtype exhibited fewer MAPK-associated gene mutations in concordance with *CIC* mutation. Additionally, mutation of *CIC* resulted in increased alterations of genes and cell signals related to tumorigenesis and MAPK activation. Because of this, mutated *CIC* can possibly be a predictor of MAPK mutation rate, pathway activation, and heightened tumorigenesis. Characterizing this relationship with MAPK through MPAS, mutant *CIC*, specifically, drove higher trending MAPK activation scores in GBM and astrocytoma, with significant increases shown in oligodendroglioma. Interestingly, the survival analyses via CODEai algorithm reported enhanced survival in patients with *CIC* mutations over patients with *CIC* wild-type. Across oligodendroglioma, astrocytoma, and GBM, patients with identified *CIC* mutations survived longer, on average, than patients without mutations. This was the case for all CODEai analyses, including those patients treated with TMZ (Supp. Figure 1) and those treated with TMZ harboring *IDH* mutations and *MGMT* methylation (Supp. Figure 2).

Utilizing inhibitory agents to block oncogenic molecular cascades from occurring have shown the most promise in glioma therapies. Four MEK/MAPK inhibitors have been approved for use in solid tumors: trametinib, binimetinib, selumetinib, and cobimetinib [41–43]. Each have been evaluated in clinical cohorts and have yielded results supporting use in both pediatric and adult populations with MAPK aberrations, adding to the repertoire of available therapies outside of TMZ [43–46]. These results exemplify the ability of these therapeutics to penetrate the blood brain barrier effectively and should be further studied to evaluate response variation in patients with MAPK-related genetic variation.

Though the findings mentioned do lend insight into the difficulties that are seen with treatment of glioma, it was describing the relationship between *CIC* and MAPK that elucidate on treatment efficacy and survivability concerns that drive the significance home. While a significant amount of attention is being given toward immunotherapies in many other solid tumors, the ‘cold’ TME of glioma, with no correlative relationship to *CIC* status (Supp. Figure 1), renders many immunotherapies ineffective. We have shown that mutated *CIC*, a MAPK-relevant marker, causes an increased rate of oncogenic mutations, possibly driving tumorigenesis. In addition, mutated *CIC* created increased MAPK activation significantly in oligodendroglioma. Because the largest percentage of glioma subtype *CIC* mutations are found in oligodendroglioma, this observation may help in selecting MAPK inhibitors as a therapeutic option for these patients in addition to the ability of MAPK inhibitors to cross the blood–brain barrier, overcoming a major challenge that significantly reduces the efficacy of most therapeutics [47]. Even though few significant shifts in the immune landscape were shown with *CIC* mutations, this work contributes to the understanding of genetic mutations that can harbor significant consequences of tumor progression. Future work on the *CIC*–MAPK relationship should include in vivo studies to examine the efficacy of MAPK inhibition on glioma tumors harboring *CIC* mutation, preferably using the four MAPK inhibitors currently approved for clinical use.

## Supplementary Information

Below is the link to the electronic supplementary material.

Supplementary file1 (DOCX 5459 kb)

## Data Availability

The datasets generated during and/or analyzed during the current study are available from the corresponding author on reasonable request. The deidentified sequencing data are owned by Caris Life Sciences. Qualified researchers can apply for access to these summarized data by contacting Joanne Xiu, PhD and signing a data usage agreement.
