# Supplementary material for: Capicua (CIC) mutations in gliomas in association with MAPK activation for exposing a potential therapeutic target
Source: Med Oncol. 2023 Jun 8;40(7):197. doi: 10.1007/s12032-023-02071-0 (PMC10250263; doi:10.1007/s12032-023-02071-0)
Supplement: Supplementary file 1 — Supplementary file1 (DOCX 5459 kb) [file 12032_2023_2071_MOESM1_ESM.docx]

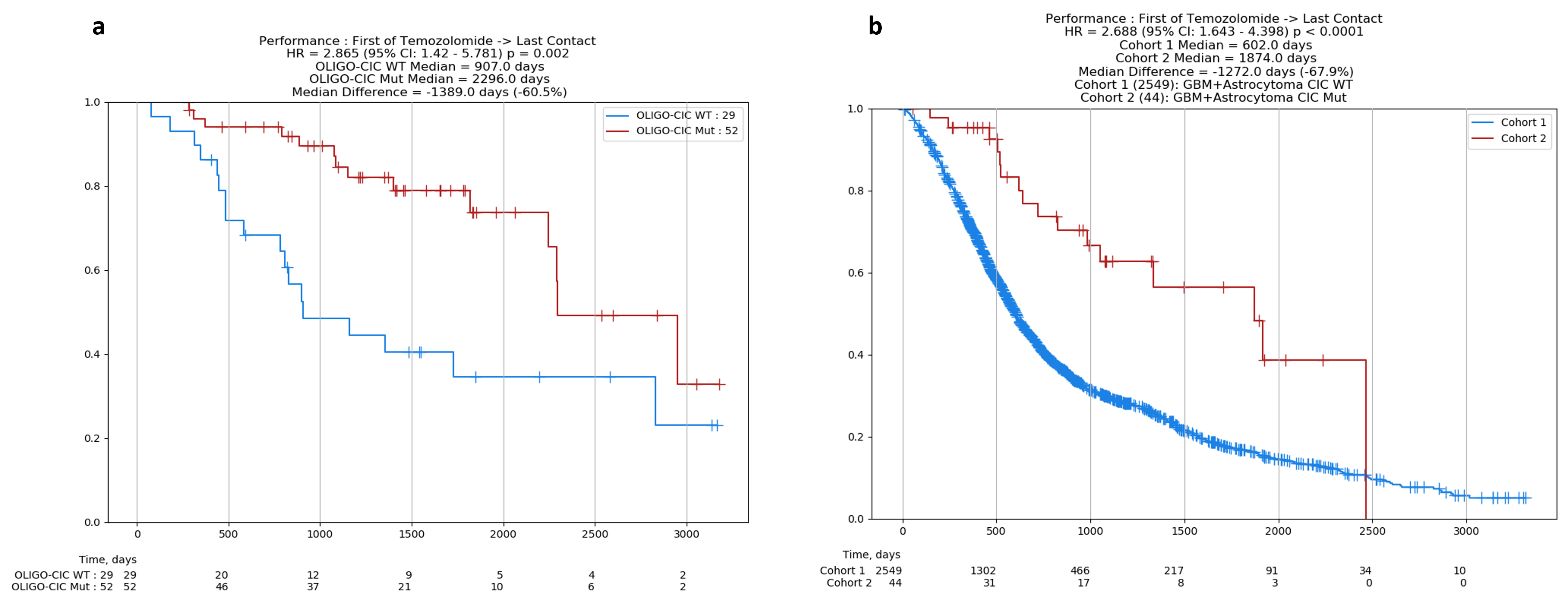


**Supplemental Fig 1 – Glioma patient survival with or without *CIC* mutations post-TMZ therapeutic regimen**CODEai survival analysis on TMZ-treated oligodendroglioma (A) and astrocytoma and GBM patients (grouped) (B) by mutated *CIC* (red) status and wild-type *CIC* (blue) status


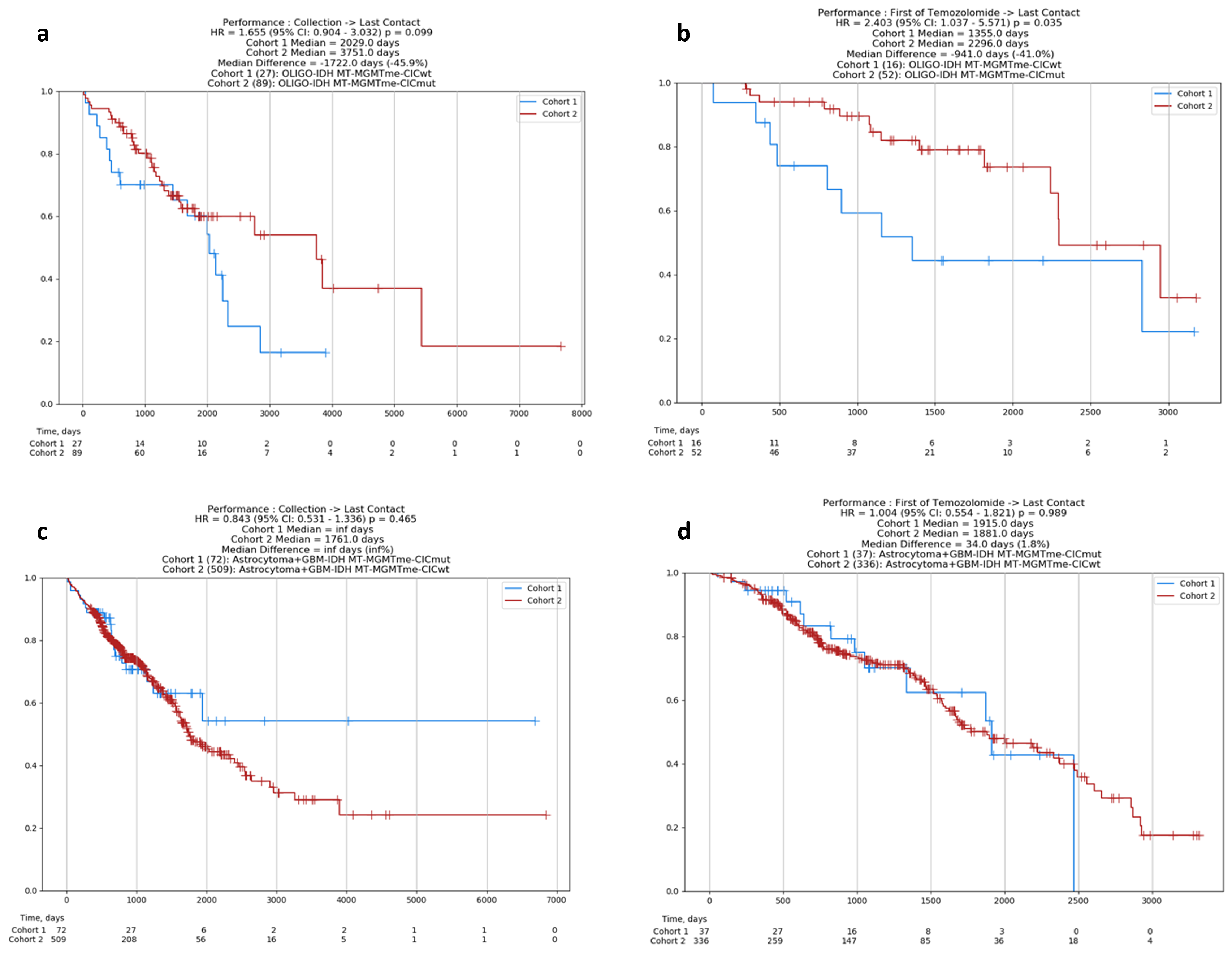


**Supplemental Fig 2 – Clinical outcome of IDH MT/MGMT-promoter methylated glioma patients with of without *CIC* mutations**CODEai survival analysis in IDH MT/MGMT-Me oligodendroglioma (A), post-TMZ treated IDH MT/MGMT-Me oligodendroglioma (B), IDH MT/MGMT-Me astrocytoma and GBM (C), and post-TMZ in IDH MT/MGMT-Me astrocytoma and GBM (D) by *CIC* mutated (red) and *CIC* wild-type (blue)


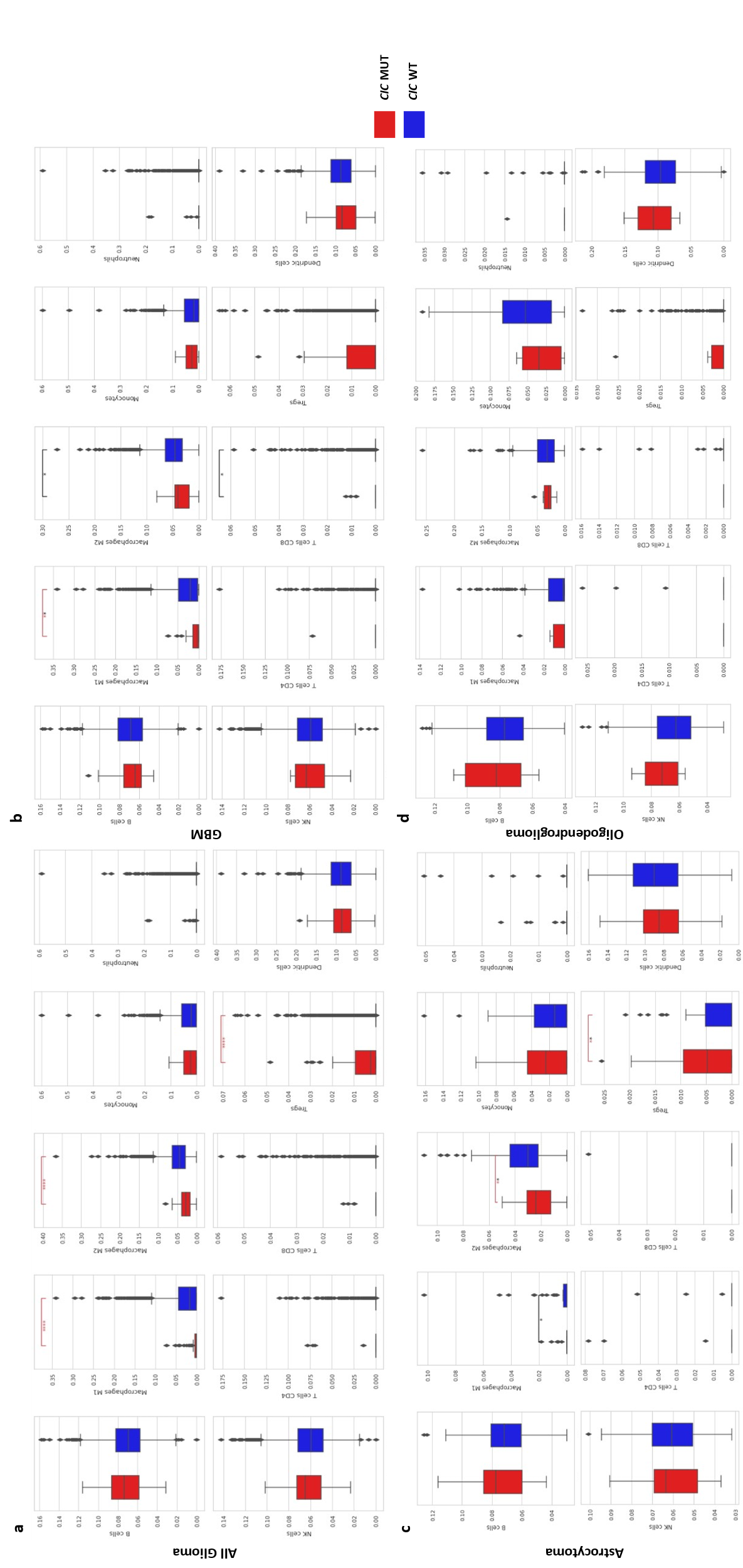


**Supplemental Fig 3 –** **Immune cell recruitment differences between *CIC* mutant and *CIC* wild-type tissue is limited to M1 and M2 macrophage, regulatory T cells, and CD8+ T cells**QuanTIseq data for all glioma (A), GBM (B), astrocytoma (C), and oligodendroglioma (D) reveal few differences in immune cell recruitment related to *CIC* mutation status. * q < 0.05, ** q < 0.01 (red). * p < 0.05, ** p < 0.01 (black)
